# Testing the Suitability of a Terrestrial 2D LiDAR Scanner for Canopy Characterization of Greenhouse Tomato Crops

**DOI:** 10.3390/s16091435

**Published:** 2016-09-06

**Authors:** Jordi Llop, Emilio Gil, Jordi Llorens, Antonio Miranda-Fuentes, Montserrat Gallart

**Affiliations:** 1 Department of Agrifood Engineering and Biotechnology, Polytechnic University of Catalonia, Esteve Terradas, 8, Castelldefels 08860, Spain; jordi.llop-casamada@upc.edu (J.L.); Montserrat.gallart@upc.edu (M.G.); 2 Department of Rural Engineering, Area of Rural Mechanization and Technology, University of Cordoba, Córdoba 14005, Spain; ir2llcaj@uco.es (J.L.); antonio.miranda@uco.es (A.M.-F.)

**Keywords:** greenhouse, tomato crop, LiDAR sensor, canopy characterization, Leaf Area Index (LAI)

## Abstract

Canopy characterization is essential for pesticide dosage adjustment according to vegetation volume and density. It is especially important for fresh exportable vegetables like greenhouse tomatoes. These plants are thin and tall and are planted in pairs, which makes their characterization with electronic methods difficult. Therefore, the accuracy of the terrestrial 2D LiDAR sensor is evaluated for determining canopy parameters related to volume and density and established useful correlations between manual and electronic parameters for leaf area estimation. Experiments were performed in three commercial tomato greenhouses with a paired plantation system. In the electronic characterization, a LiDAR sensor scanned the plant pairs from both sides. The canopy height, canopy width, canopy volume, and leaf area were obtained. From these, other important parameters were calculated, like the tree row volume, leaf wall area, leaf area index, and leaf area density. Manual measurements were found to overestimate the parameters compared with the LiDAR sensor. The canopy volume estimated with the scanner was found to be reliable for estimating the canopy height, volume, and density. Moreover, the LiDAR scanner could assess the high variability in canopy density along rows and hence is an important tool for generating canopy maps.

## 1. Introduction

Public concerns due to environmental problems associated with an inaccurate pesticide application process led the European Directive 2009/128/EC of the European Parliament to establish a regulatory framework [1]. In this document, the need to improve the efficiency in the use of Plant Protection Products (PPPs) is remarked. For this purpose, pesticides dose must be adjusted according to the canopy characteristics, thus avoiding overdosing and unnecessary losses to the environment.

The greenhouse tomato crop, grown to be consumed as a fresh product, is very important in Spain, with a cultivated area of 6189 ha [2]. The accurate application of pesticides is essential for all type of crops or circumstances. In particular, fresh products to be directly commercialized in the market require accurate and safe pesticide application in order to prevent health risks. Pesticide residues on vegetables constitute a possible risk to consumers and have been a human health concern [3]. However, although some researchers have evaluated the optimal volumes of pesticides to be applied [4,5], few studies have related all parameters influencing the relationship between the canopy characteristics and the amount of plant protection product according to the real needs.

Greenhouse tomato rises from the ground and develops a long stem, which is fixed by the farmer to a fixed structure to make it stay in a vertical disposition. Therefore, this crop belongs to the group called 3D crops; that is, crops that present a complex geometry for the sprayer in contrast to arable crops, which are treated as if they were a flat 2D target. The constant dose per unit ground area results less suitable for 3D crops [6], because the varying geometry of the vegetation make it difficult to set a general application volume that results in a satisfactory application quality. Therefore, researchers have established other systems that focus on different parameters related to the canopy structure. The first two methodologies proposed were the Tree Row Volume (TRV) and Leaf Wall Area (LWA). The TRV method involves calculating the canopy volume by assuming its prismatic shape; hence, the canopy height and width, along with the row spacing, are the base parameters to determine the TRV, which is expressed in cubic meter canopy per hectare of ground [7,8]. The application volume will be proportional to this TRV parameter according to a specific coefficient that will have different values according to the crop [9,10,11]. On the other hand, the LWA is calculated based on the assumption that the canopy sides are completely flat, and hence, they form a “wall”. Canopy height is the main parameter to calculate the LWA [12], and therefore, the canopy width is ignored. The LWA is expressed in square meters of LWA per hectare of ground. The sprayed dose is calculated for every 10,000 m^2^ of LWA. These two systems are well-established, and at present, there is a general discussion among the countries of the European Union regarding which of these systems should be used as the standard label dosing system for all crops [13,14]. Nevertheless, in recent years, researchers have proposed alternative systems because the TRV and LWA methods do not consider the leaf density, which is an important canopy parameter [14]; therefore, these methods are incomplete. Various dosing systems have been proposed for different crops, including vineyards and citrus and fruit trees such as apple trees [6,15,16,17,18]. Although these systems differ in their basis, assumptions, and calculations, they all rely on an accurate canopy characterization system.

Various methods for canopy characterization, which is a complex task, have been proposed in the last years. The canopy characterization methods can be classified in two general categories: manual and electronic methods. The manual methods are based on manual measurements with a measuring tape or topographic milestone. These methods vary according to the canopy structure and are much simpler for hedgerow orchards than for isolated trees or plants. Although they are reliable, fast, and simple to use for the farmer, they become less useful for more advanced tasks such as generating prescription maps for proportional spray application, like the one proposed by the aforementioned dosing systems. In addition, it is difficult to evaluate the canopy density with manual methods because they require complete defoliation of a representative plant sample to obtain reliable values. Therefore, the electronic methods seem to be an appropriate option to accomplish the requirements of dose adjustment. Electronic characterization methods using ultrasonic sensors [18,19,20,21], stereo vision [22], light sensors [23], and LiDAR scanners [24,25,26,27,28,29] are more frequently used. According to Rosell and Sanz [30], LiDAR is the most accurate technology for canopy characterization, and in fact, it has been demonstrated to be very reliable at predicting canopy parameters in different studies [20,24,31]. The LiDAR scanner uses the time-of-flight principle to calculate distances—the sensor measures the elapsed time between laser beam emission and reception and automatically calculates the distance to the target point [32]. This process is repeated along a plane in 2D scanners or in three dimensions by rotating the scanning plane in 3D LiDAR. The 2D sensor is cheaper and can have a third coordinate by moving it along the axis perpendicular to the scanning plane [24,28]; hence, it is more frequently used for canopy characterization.

The characteristics of tomato plants—thin, tall, and planted in pairs—make their characterization with the electronic methods difficult because it is difficult to identify the parameters related to each individual plant. Furthermore, the narrow row spacing limits the field-of-view of the sensors used. Therefore, this study aims to: (1) assess the accuracy of the LiDAR sensor for determining major canopy parameters related to canopy volume and density; (2) establish useful correlations between manual and electronic parameters for leaf area estimation; and (3) exploit the LiDAR technology to assess the variation in canopy density along a row as a basis to generate canopy density maps for pesticide dose adjustment.

## 2. Materials and Methods

### 2.1. Experimental Fields

**Figure 1:**
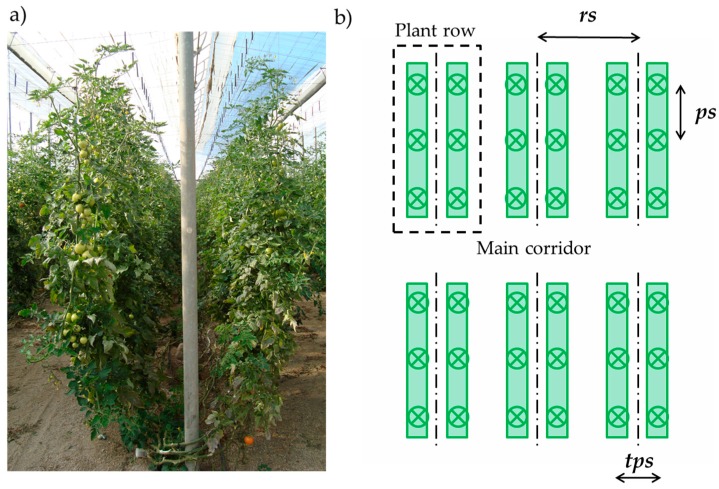
(**a**) Twin plantation system; (**b**) Plantation layout inside the greenhouse, with row spacing, *rs*, plant spacing in a row, *ps*, and twin plant spacing, *tps*.

The experiments were performed in three different tomato cultivar greenhouses located in El Ejido (Almería, Spain) (36°45′22.90′′ N; 2°48′34.89′′ W) and Viladecans (Barcelona, Spain) (14°18′46.46′′ N; 2°1′48.44′′ W), both important fresh produce growing areas on the Spanish Mediterranean coast. The greenhouses grew tomato crops of the *Velasco* and *Barbastro* varieties with similar plantation patterns (Table 1). The plants were planted in a twin row system (Figure 1a), where the crop was planted in pairs in the same row. The three greenhouses had a main corridor with adjacent and perpendicular rows (Figure 1b). The row spacing, *rs*, plant spacing in the row, *ps*, and twin plant spacing, *tps*, are specified in Table 1 and represented in Figure 1b.

### 2.2. Manual Canopy Characterization

**Figure 2:**
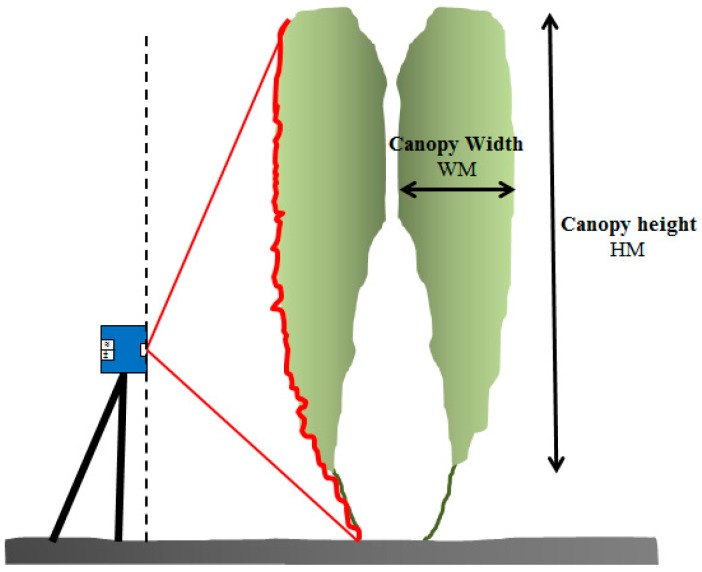
Measured parameters for the manual canopy characterization and LiDAR scanner location.

For manual canopy characterization, the total canopy height, H_M_, and canopy width, W_M_, were measured along the row. The measurements were performed with a measuring tape by the same operators in the three fields of study, with 30 replications per field of study for each measurement. The total canopy height, H_M_, was measured from the lowest leaves on the plant stem to the top leaf of each plant (Figure 2). The canopy width was measured from the outer to the inner part of the canopy. The measurement was done at 1.5 m of the plant height as a compromise to the thicker part of the plant and the wider part. Each plant of the twin plantation system was measured separately (Figure 2).

The total leaf area per single plant was also determined. The plants were collected in pairs: two pairs (four plants) for greenhouses 1 (GH1) and 2 (GH2) and three pairs (six plants) for greenhouse 3 (GH3). They were appropriately stored in sealed plastic bags. Then, under laboratory conditions and before they had dried out, the leaves were removed from the plants and subsamples 80 g in weight were planimetered with a leaf planimeter (LI 3100C, LI-COR, Lincoln, NE, USA) to obtain the total leaf area of the subsample (cm^2^) as well as the leaf area–weight ratio [4,33,34], which enables obtaining the leaf surface area by only weighing the leaves, thus saving time.

From these measured parameters, the other parameters could be calculated: TRV that quantify the amount of canopy volume per ground surface from canopy height and width and the distance between rows, data is expressed in cubic meters per hectare of ground [7,33,35]; LWA that quantify the canopy surface per ground surface from canopy height and the distance between rows, data is expressed in square meters of vegetation per hectare of ground [36,37]; Leaf Area Index (LAI) that shows a dimensionless ratio between leaf area and ground area surface; and Leaf Area Density (LAD) obtained from the LAI and TRV values expressed as square meters of vegetation divided by cubic meters of canopy [38,39].

### 2.3. LiDAR Canopy Characterization

#### 2.3.1. Canopy Scanning

A terrestrial 2D low-cost general-purpose LiDAR scanner (LMS-200, Sick, Düsseldorf, Germany) was used in this study. It is a fully automatic divergent laser scanner that can measure time-of-flight with an accuracy of ±15 mm in a single shot measurement and a 5 mm standard deviation in a range up to 8 m [20]. The sensor has a maximum scanning angle of 180° and selectable angular resolutions of 1°, 0.5°, and 0.25°. A scanning angle of 180° has been shown to be suitable for accurate canopy characterization [40]; therefore, it was chosen for the present study. The device was supplied with 24 V by an autonomous battery and it was connected to a laptop via an RS-232 serial port for data transmission.

The sensor was installed at the centre of the space between the crop rows and it was mounted opposite to the canopy in such way that it can properly scan the entire plant from the base to the top (Figure 2). The sensor was then moved along a constant track, scanning the pair of plants from both sides. Although the same plant could not be scanned from both sides because of their paired disposition, the high resolution of the scanner enabled a high percentage of the laser beams to penetrate the first plant and scan the second. Furthermore, three replications per side and canopy section were performed.

**Figure 3:**
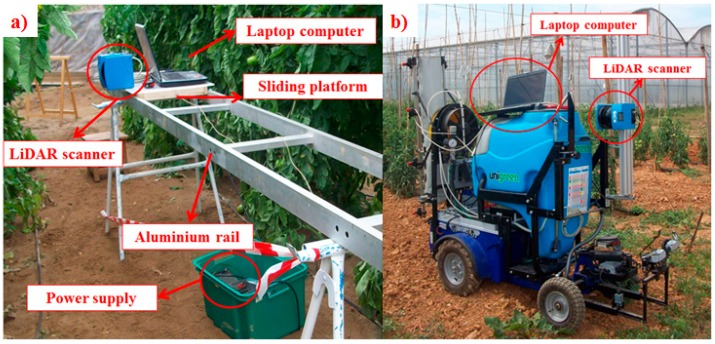
(**a**) Fixed structure of the LiDAR support system for measurements in greenhouses 1 and 2; (**b**) LiDAR scanner mounted on a radio-controlled mobile platform for measurements in greenhouse 3.

Two types of structures were used in the scanning process. In GH1 and GH2, the LiDAR sensor was mounted on a mobile platform that was manually pulled at a constant average speed (0.06 m·s^−1^ ± 0.009) to make it slide along an aluminium rail 2.4 m in length mounted on trestles (Figure 3b). In GH3, the LiDAR sensor was mounted on an autonomous spraying platform described in Balsari et al. [41] (Figure 3c). This platform was moved by an electric engine and remotely radio controlled. In both cases, the data-acquiring laptop was mounted on the platform to simplify the wiring connections.

The mobility of the autonomous platform in GH3 enabled scanning the entire tomato row (23.4 m in length) from both sides of the canopy with three replications. These measurements enabled obtaining information regarding canopy variation along the row.

#### 2.3.2. Data Processing

**Figure 4:**
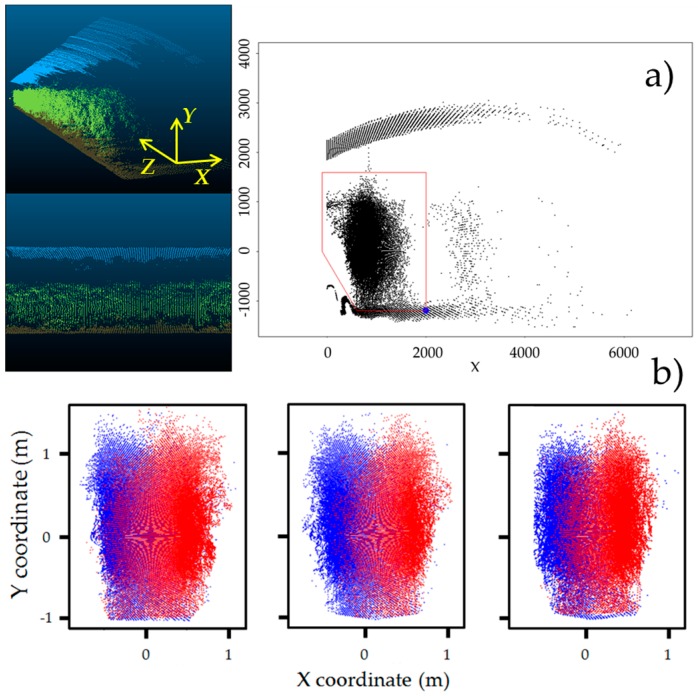
(**a**) LiDAR points cloud from one side in CloudCompare^®^ software with coordinate system and canopy delimitation procedure; (**b**) Plant delimitation process from twin plants (three replications).

Data from the LiDAR sensor was obtained in polar coordinates (each point has an angle direction and distance response). To manage the information, the raw data was converted to *XYZ* coordinates with R-software^®^ (3.0.2) (R Development Core Team, 2013, Vienna, Austria), where *X* axis corresponds to the plant width, *Y* axis is the plant height, and *Z* axis is the row length (Figure 4a).

Because the LiDAR sensor was mounted on two different structures for the measurements, the analysed values could have variations. Furthermore, the forward speed of the sensor varied among replications (coefficient of variation 16.8%) in the case of the fixed structure as it was manually driven. Therefore, number of LiDAR scans were normalized by considering the forward speed of the mobile sensor and the scanning frequency (Hz). This speed could be calculated in the analysis process because the data acquisition system recorded the time elapsed since the beginning of data recording and the LiDAR track’s length was known. Assuming these differences, a fixed length of canopy to be evaluated was stablished. Then, the number of slices of LiDAR measurements to be analysed were determined for every single replication in order to evaluate the same length of canopy.

Once the data were appropriately normalized, the results were imported to the CloudCompare^®^ software (TelecomParisTech, Paris, France) in order to obtain the 3D LiDAR points cloud and to ensure that there were no problems or irregularities in the data acquisition process or data normalization. As the LiDAR sensor does not only scan the plants but also scans the greenhouse’s top and ground as well as the sensor support system, the points that belong to the canopy must be defined and distinguished from the others. This process was performed for each scanning file (from one side) by observing the points cloud from the *Z* axis with an orthographic projection and determining some border points by setting one of the known coordinates and obtaining the remaining from the first (Figure 4a). Then, both sides of the scanned plants were manually aligned and positioned to define the entire canopy structure (Figure 4b). After this first approach, it was necessary to delimit the points belonging to each one of the two paired plants (Figure 4b). This process was performed manually by determining their centre, which was assigned as the (0,0) coordinate.

At this stage, different parameters, such as canopy height, H_L,_ and width, W_L_, the number of points on the target (IMP), and the canopy volume, V_L_, could be obtained or calculated from the LiDAR points cloud.

To calculate H_L_, the difference between the highest and lowest points in each LiDAR slice (Figure 4a), i.e., the maximum length on the *Y* axis for each LiDAR profile, was determined. H_L_ was then calculated as 95% of the maximum value among all previously determined values. This 95% value was chosen to filter possible unusual profiles or data errors that could affect the measurement reliability. W_L_ was calculated by determining half of the total width, measured on the *X* axis, of each plant pair. Once this distance was known, W_L_ was obtained as 95% of the value for the aforementioned reasons.

IMP was determined as the number of LiDAR beam impacts on the canopy per row length unit (impacts m^−1^). This parameter was included in the analysis process owing to its significant correlation with manually measured LAI values in a previous study performed in a vineyard [20].

To obtain the canopy volume per single plant, V_L_, the methodology described in Xu et al. [42] and in Miranda-Fuentes et al. [40] was applied. This methodology divides the points cloud corresponding to the entire canopy into horizontal slices of a certain height, Δ*h*. Next, all points belonging to the same slice are projected on the same horizontal plane. Then, their external perimeter is delimited using the convex hull algorithm [43], and its inner area, A_i_, is determined. The volume of each slice, V_L_, can be calculated as its internal area, A_i_, multiplied by its height, Δ*h*. Therefore, the total volume of the plant is calculated as:

(1) $$V_{L}=\sum_{i=1}^{n}A_{i}\times \Delta h,$$

where *n* is the number of horizontal slices, *V_L_* is expressed in cubic meters, *A_i_* in square meters, and Δ*h* in meters.

As it is evident, the lower the Δ*h*, the higher the vertical resolution of the method. In some studies, Δ*h* values of 0.001 m have been used [42]. Nevertheless, values of 1 cm have been shown to be sufficiently accurate in previous studies [40] and to accelerate the calculation process. Therefore, we chose a Δ*h* value of 0.01 m in the present study.

### 2.4. Statistical Analysis

In the statistical analysis, a linear correlation between all measured and calculated parameters was performed using the statistical R-Software^®^ (3.0.2) (R Development Core Team, 2013) with the *Agricolae* package. The data analysis related all measured and calculated results to identify the most significant and interesting correlations between them, always considering the manually measured parameters as a reference.

The Shapiro-Wilk test (*p* > 0.05) [44,45] and a visual inspection of the data histograms were performed. Moreover, normal Q-Q plots and box plots were drawn to ensure that the data were normally distributed in all cases. The interest of the linear correlations between the parameters obtained from the manual characterization, H_M_, W_M_, LAI, TRV, LAD, and LWA, and those obtained from the LiDAR scanning of plants, H_L_, W_L_, V_L_, and IMP was evaluated with the correlation p-values and their determination coefficients (R^2^).

## 3. Results

### 3.1. Canopy Characterization Parameters

The parameters obtained from the canopy characterization are listed in Table 2. It can be observed that the canopies of the three greenhouses had similar height characteristics. The maximum height of the plants is not determined by the plant growth but by the structure of the greenhouse, in which the stems are fixed to the greenhouse structure when they grow to that level, continuing the growth process downwards toward the ground. The canopy width is quite different overall in GH2, which also has a low LAD. Note that the width values were measured from the centre of the two paired plants to the edge of each plant. These two parameters, especially the height, were constant in all studied fields.

The lowest value of TRV is found in GH2 (7771 m^3^·ha^−1^), which is significantly different from those in GH1 and GH3 (10,882 and 10,397 m^3^·ha^−1^, respectively). These differences can be explained by the difference in the measured canopy width. Therefore, the LWA did not follow the same trend as the TRV; it was the largest in GH3, at 39,170 m^2^ ha^−1^, and had very similar values in GH1 and GH2.

The LAD was the lowest in GH2 (3.15 m^2^·m^−3^) and very similar in the other two fields (5.81 and 5.30 m^2^·m^−3^ in GH1 and GH3, respectively).

Regarding the electronically measured parameters, the LiDAR height, H_L_, was found to be generally lower than that manually measured, H_M_, with a 12.12% lower mean value. Nevertheless, the H_L_ parameter followed a trend similar to H_M_, with the maximum height being measured in GH2. On the other hand, the canopy width was overestimated by the scanner, but this mainly occurred in the case of GH2, in which the electronically measured canopy width was 48% greater than the manually measured value.

The standard errors of the mean (SEM) in the measurements are generally low, being below 10% in all cases and below 1% in most cases. The standard errors in the geometrical measurements of all parameters of the three GHs are very similar. The standard error in the measurement of the LAD parameter is slightly higher, which is normal considering the variability of this parameter along the canopy.

### 3.2. Correlations among Parameters Obtained with Manual and Electronic Methodologies

Table 3 shows the determination coefficients (R^2^) for all paired linear correlations among all parameters related to the canopy volume and density.

The height (H_L_) parameter obtained with the LiDAR has been significantly correlated with the manually measured height, H_M_ (R^2^ = 0.59), manual width, W_M_, (R^2^ = 0.52), and manual TRV value (R^2^ = 0.46). Nevertheless, there is no correlation between H_L_ and LWA (R^2^ = 0.004). This could be because this parameter was not proportional to the canopy height in the three GHs and was the maximum in GH3 even when the maximum height was found in GH2 (Table 2).

On the other hand, the LiDAR width, W_L_, was only significantly correlated with the LWA; even the LWA calculation is not affected by the canopy width; this correlation shows the importance of the width in these types of crops where the height is limited by the greenhouse structure.

The LiDAR volume, V_L_, seems to be the most reliable parameter to estimate the geometrical characteristics of the canopy as it is significantly correlated with the H_M_, TRV, and LWA with determination coefficients of 0.69, 0.37, and 0.33, respectively. It can be observed that the determination coefficients of the TRV and LWA are very similar. Because the LiDAR volume, V_L_, is statistically reliable, it could be the most complete parameter for estimating the TRV and LWA.

All correlations between the canopy density parameters—LAI and LAD—and the other parameters are presented in Table 3. Interesting correlations can be observed between some manually measured geometrical parameters, such as H_M_ and W_M_, and the canopy density. In fact, both parameters are significantly related to the LAI (R^2^ = 0.60 and R^2^ = 0.70 for H_M_ and W_M_, respectively), and to the LAD (R^2^ = 0.53 and R^2^ = 0.65 for H_M_ and W_M_, respectively), which is not surprising as both density parameters are closely related. The TRV values are highly correlated to the LAI and LAD values with determination coefficients of R^2^ = 0.89 and R^2^ = 0.79, respectively. On the other hand, the LWA values were found to not be appropriate estimators of the leaf density, showing no significant correlations. The IMP parameter, expressed as the number of LiDAR impacts per length unit, has been shown to have strong correlations with the leaf density parameter in previous studies. In this study, IMP was found to be inaccurate for predicting the LAI and LAD values of tomato plants. More tests need to be performed to identify the reason for this.

**Figure 5:**
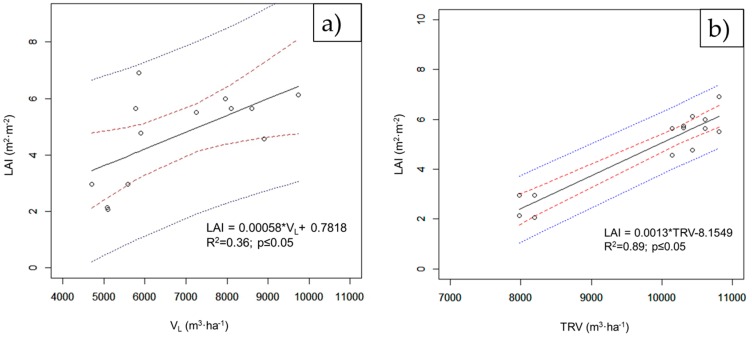
Linear correlations between (**a**) LAI and V_L_ and (**b**) LAI and TRV.

Figure 5 shows the correlations between the LAI and V_L_ (Figure 5a) and those between the LAI and TRV (Figure 5b). It can be seen that the TRV values are well aligned with the LAI values. On the other hand, V_L_ has a lower determination coefficient, R^2^ = 0.36.

### 3.3. Canopy Characterization Along a Row Based on LiDAR Scanner Measurements

**Figure 6:**
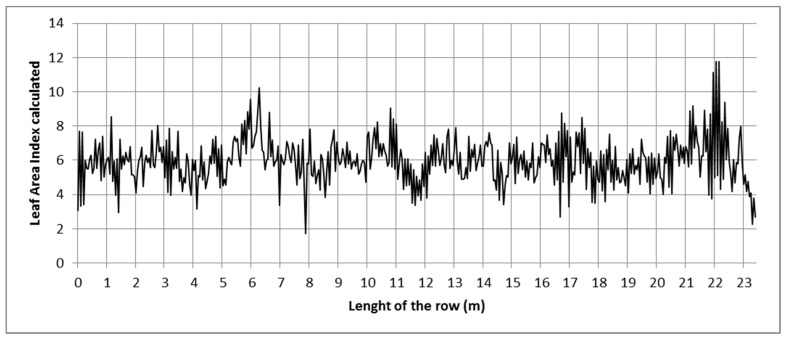
Calculated LAI variation along the scanned row in GH3.

The mobile platform enabled scanning the entire row from two sides. The LAI was used as an example of the variation in the vegetation along the row. This estimation was based on the V_L_ as it was found to be the most accurate with the largest determination coefficient among the studied parameters. The calculated variation in the LAI in GH3 is shown in Figure 6. In this graph, the variation in the LAI value is calculated every 10 cm.

Although the variation range is relatively constant along the row, the continuous changes in the canopy reflect the important variation in the LAI. The LAI values usually range from 3 to 9, with exceptions like those found for the *Z* positions 6 m, 8 m, and 22 m. The variation rate, calculated as the number of times the LAI value varies by more than 10% per linear meter, has a mean value of 10 m^−1^. The values observed in Figure 6 are consistent with the LAI mean value (5.9). The standard error was found to be very small (0.17) in the manual measurements.

## 4. Discussion

A 2D LiDAR scanner was used to electronically obtain canopy parameters related to the canopy volume and density of a 3D crop with a complex structure, which is a difficult task. The general results in Table 2 show that the LiDAR values for geometrical characteristics, such as height and width, differ from the manual measurements, which were overestimated. This has also been observed in previous studies using this sensor [20,40]. The plant height value is influenced by the manual measurement method, in which one operator stands with a topographic milestone and other, at a certain distance, must take the measurements by observing the top part of the plants. As this height is important (>2 m) and the row spacing is narrow (2–2.8 m), the operator must have good skills in reading the height value and must not instead read its conical projection. In the case of the width, the most external points are taken, and therefore, the measured width for each section is not the mean but the maximum.

It is very noticeable the fact that the mean LWA values in the three GHs do not coincide with the H_M_ values, with the maximum mean value observed in GH3 rather than in GH2, which has the highest mean H_M_ value. At this point, the row spacing has a greater influence on the LWA calculation than the canopy height. On the other hand, the TRV values show a similar behaviour related to variation in the height and width values. In this particular case, the obtained data show that because the canopy height is constant (because of the greenhouse structure) and the row spacing is also determined by the farmer and conditioned by the greenhouse structure, the only parameter that changes is the canopy width. Therefore, in the case presented in this research, the TRV method seems to be more suitable than the LWA method to determine the canopy volume and density, which are mainly influenced by the row spacing.

In greenhouse tomato crops, the evolution of the LAI is linked to the plant height until the plant reaches the top of the greenhouse structure, where the canopy grows along the width. In this case, the TRV seems more suitable to describe the vegetation because it gives more information across the canopy width rather than the LWA, which in this particular case, is more affected by row spacing than by canopy height.

To estimate the canopy volumes, given by its TRV, it could be said that the LiDAR methodology is an interesting alternative measurement procedure, with acceptable determination coefficients, especially for H_L_ (Root Mean Square Error (RMSE) 9659.4 m^3^·ha^−1^) and V_L_ (RMSE 3446.09 m^3^·ha^−1^). This has been observed in other crops such as vineyards [20], hedgerow fruit trees [46], and large isolated trees like citrus [47] or olive [40]. Spray application based on canopy volume has been shown to be sufficiently accurate to be considered a first step in the dose adjustment process even for complex canopy structures [11,48]. Therefore, it is essential to accurately estimate parameters that allow farmers or technicians to have a very simple criterion to adjust the sprayed volumes, which can be easily done by constructing a canopy volume map or using a sensor operating real time and automatically adjusting the spraying parameters [49].

The importance of canopy density has been strongly suggested by different authors for modifying the spray volume calculated with volume-based dosing methods [16,18,50]. This parameter can be automatically estimated with the LiDAR scanner, as shown by the significance of the correlations between the LAI and H_L_ and those between the LAI and V_L_. These results are consistent with those of other studies [51]. They have an important consequence in the automatic adjustment of the spray dose because the estimation of canopy density can be added to the volume estimator for the real-time adjustment of the spray dose, which has been implemented in other crops [50,52]. It was surprising that the number of LiDAR points per row length unit was not correlated with canopy density. This can be explained by the paired plantation system, which only allows the laser to scan one plant side and difficult the penetration of the laser beam into the canopy, and therefore, did not allow the researchers to properly study the correlation between the LiDAR points and the individual plant’s LAD. In further studies, this parameter should be studied from the top view in addition to the side view in order to validate this parameter.

Regarding the canopy variation along the row, the LiDAR scanner properly characterized all longitudinal variations in this parameter, and considering that this parameter can vary 10 times per meter, as a mean value, manual methods cannot handle such a high variability. In this sense, the research on mapping methodologies has been very important in recent years [30], and further research is necessary to adapt these methodologies to the particular case of paired plantation systems in greenhouse tomato crops. The optimal spray volumes should also be adjusted according to the canopy volume and density in order to transform these volume or density maps in spray volume maps to optimize the spray application process.

## 5. Conclusions

Canopy characterization with a terrestrial 2D LiDAR scanner was performed in a paired plantation system in three tomato crop greenhouses and its accuracy was compared with manual characterization methods. The following conclusions can be drawn:

- The LiDAR scanner underestimates certain manual values, but this can be due to the inherent higher resolution (larger number of point measurements) when compared with manual methodology.
- Volume parameters, such as the TRV and LWA, can be estimated with the laser scanner with a high statistical significance and high determination coefficients. This is very important to satisfy the new requirements for dose harmonization according to these parameters in the European Union to ensure the most optimal dose rate adjustments.
- LAI can be estimated by the sensor from the calculated height or volume, but not from the number of impacts per hedgerow length unit, as expected. Further improvements in the laser scanning process could improve this estimation.
- Canopy variations along a single row are very important to determine the exact input needed in each part of the field, and therefore, manual methods are unsuitable because of their low longitudinal resolution. LiDAR scanners can adapt to this variability and hence are an appropriate alternative for generating canopy density maps.

## Figures and Tables

**Table 1:** Main characteristics of the experimental fields.

Greenhouse ID	Location	Plant Layout (Row Spacing × Plant Spacing) (m × m)	Crop	BBCH Scale
GH 1	El Ejido (Almería)	2.5 × 0.4	*Solanum lycopersicum* L. cv. Velasco	79
GH 2	El Ejido (Almería)	2.8 × 0.4	*Solanum lycopersicum* L. cv. Velasco	79
GH 3	Viladecans (Barcelona)	2.0 × 0.4	*Solanum lycopersicum* L. cv. Barbastro	76

**Table 2:** Average measured and calculated geometrical and density parameters and its Standard Deviation of the MEAN.

	Parameter		Greenhouse ID		
			1	2	3
**Manual characterization**	**Manual Height**	**H_M_** (m)	2.19 ± 0.02	2.50 ± 0.02	1.96 ± 0.04
	**Manual Width**	**W_M_** (m)	0.62 ± 0.02	0.43 ± 0.04	0.53 ± 0.01
	**Tree Row Volume**	**TRV** (m^3^·ha^−1^)	10,882 ± 397	7711 ± 212	10,397 ± 252
	**Leaf Wall Area**	**LWA** (m^2^·ha^−1^)	35,111 ± 360	35,683 ± 290	39,170 ± 755
	**Leaf Area Density**	**LAD** (m^2^·m^−3^)	5.81 ± 0.28	3.15 ± 0.15	5.30 ± 0.19
**Electronic characterization**	**LiDAR Height**	**H_L_** (m)	1.90 ± 0.07	2.12 ± 0.01	1.93 ± 0.03
	**LiDAR Width**	**W_L_** (m)	0.71 ± 0.02	0.64 ± 0.02	0.59 ± 0.03
	**LiDAR Volume**	**V_L_** (m^3^)	1.13 ± 0.07	1.32 ± 0.03	2.42 ± 0.12

**Table 3:** All possible comparisons among all measured and calculated parameters related to the canopy volume and density.

			Manual Measurements						LiDAR Measurements			
			H_M_	W_M_	LAI	TRV	LWA	LAD	IMP	H_L_	W_L_	V_L_
			(m)	(m)	(m^2^·m^−2^)	(m^3^·ha^−1^)	(m^2^·ha^−1^)	(m^2^·m^−3^)	(m^−1^)	(m)	(m)	(m^3^)
**Manual**	**H_M_**	(m)	1	0.29 **	0.60 **	0.50 **	0.21 *	0.53 **	0.20 *	0.59 **	0.003	0.69 **
	**W_M_**	(m)		1	0.70 **	0.86 **	0.01	0.65 **	0.20 *	0.52 **	0.10	0.16
	**LAI**	(m^2^·m^−2^)			1	0.89 **	0.02	0.97 **	0.01	0.52 **	0.01	0.36 **
	**TRV**	(m^3^·ha^−1^)				1	0.08	0.79 **	0.03	0.46 **	0.01	0.37 **
	**LWA**	(m^2^·ha^−1^)					1	0.01	0.51 **	0.004	0.29 **	0.33 **
	**LAD**	(m^2^ m^−3^)						1	0.01	0.47 **	0.01	0.32 **
**LiDAR**	**IMP**	(m^−1^)							1	0.0001	0.17	0.27 *
	**H_L_**	(m)								1	0.10	0.31 **
	**W_L_**	(m)									1	0.03
	**V_L_**	(m^3^)										1

Selection criteria: * interesting relationship; ** good correlation is expected.
